# Comparison of the Osteogenic Potential of Titanium and Modified Zirconia-Based Bioceramics

**DOI:** 10.3390/ijms15034442

**Published:** 2014-03-13

**Authors:** Young-Dan Cho, Ji-Cheol Shin, Hye-Lee Kim, Myagmar Gerelmaa, Hyung-In Yoon, Hyun-Mo Ryoo, Dae-Joon Kim, Jung-Suk Han

**Affiliations:** 1 Department of Prosthodontics, School of Dentistry and Dental Research Institute, BK21 Program, Seoul National University, Seoul 110-749, Korea; E-Mails: cacodm@hanmail.net (Y.-D.C.); sjcyj@snu.ac.kr (J.-C.S.); kim.haley12@gmail.com (H.-L.K.); myagmar.gerelmaa@gmail.com (M.G.); prosthoyoon@gmail.com (H.-I.Y.); 2 Department of Molecular Genetics, School of Dentistry and Dental Research Institute, BK21 Program, Seoul National University, Seoul 110-749, Korea; E-Mail: hmryoo@snu.ac.kr; 3 Department of Advanced Materials Engineering, Sejong University, Seoul 143-747, Korea; E-Mail: djkim@sejong.ac.kr

**Keywords:** dental implant, titanium, zirconia, LTD, osteogenic potential

## Abstract

Zirconia is now favored over titanium for use in dental implant materials because of its superior aesthetic qualities. However, zirconia is susceptible to degradation at lower temperatures. In order to address this issue, we have developed modified zirconia implants that contain tantalum oxide or niobium oxide. Cells attached as efficiently to the zirconia implants as to titanium-based materials, irrespective of surface roughness. Cell proliferation on the polished surface was higher than that on the rough surfaces, but the converse was true for the osteogenic response. Cells on yttrium oxide (Y_2_O_3_)/tantalum oxide (Ta_2_O_5_)- and yttrium oxide (Y_2_O_3_)/niobium oxide (Nb_2_O_5_)-containing tetragonal zirconia polycrystals (TZP) discs ((Y, Ta)-TZP and (Y, Nb)-TZP, respectively) had a similar proliferative potential as those grown on anodized titanium. The osteogenic potential of MC3T3-E1 pre-osteoblast cells on (Y, Ta)-TZP and (Y, Nb)-TZP was similar to that of cells grown on rough-surface titanium. These data demonstrate that improved zirconia implants, which are resistant to temperature-induced degradation, retain the desirable clinical properties of structural stability and support of an osteogenic response.

## 1. Introduction

Several types of biomaterials have been used in dental implant studies; among them, titanium has been considered the most useful, as it has excellent mechanical properties and biocompatibility [1,2]. Modification of titanium surfaces via different additive (bioactive coatings) and subtractive processes (acid etching, grit-blasting) can improve osseointegration [3–10]. Additional trials showed that incorporation of titanium into glass-based biomaterials could enhance biological responses [11,12]. However, titanium’s metallic grayish color sometimes causes aesthetic problems in the anterior part of the dental implantation, as there is insufficient soft tissue to mask the peri-implant region. Furthermore, allergic reactions and sensitivities to titanium have been reported [13,14]. To minimize the soft tissue recession and aesthetic problems, many implant collars based on non-metallic materials have been developed. Tooth-colored and biocompatible ceramic materials or bioactive glass substrates are also potential candidates for novel implants [15]. Alumina is a highly biocompatible ceramic material with good aesthetic properties, but is associated with a high fracture risk. Because of this critical weakness, zirconia was introduced as a titanium alternative [16,17]. Zirconia exists in three phases, monoclinic (M), cubic (C) and tetragonal (T), depending on temperature. M-phase is fragile at room temperature, and therefore requires stabilization to prevent tetragonal (T)-to-monoclinic (M) phase transformation in technical applications [18,19]. A stress-induced transformation toughening mechanism improves the mechanical strength of zirconia, rendering it more suitable as a dental implant material [17,20]. Yttria (Y_2_O_3_) is used as a general stabilizer for maintaining the T-phase of ZrO_2_. Y_2_O_3_-stabilized tetragonal zirconia polycrystals (Y-TZP) have high strength, toughness, and biocompatibility, and elicit biological responses that are similar to those induced by titanium [21–23]. Therefore, Y-TZP is considered as a potential titanium alternative. However, zirconia exhibits structural instability upon low temperature degradation (LTD, often referred as “aging”), which is due to tetragonal (T)-to-monoclinic (M) phase transformation in moist or stress conditions [24]. Clearly, this limits the clinical utility of zirconia. Since the T-to-M transformation rate is most rapid at ~250 °C, it was not initially considered as a liability under physiological conditions of 37 °C [25,26]. However, several clinical failures in the use of hip prostheses were subsequently reported [25–29]. This spurred many efforts to inhibit LTD-dependent phase transformation, including addition of stabilizers such as niobium oxide (Nb_2_O_5_) [30,31] or tantalum oxide (Ta_2_O_5_) [32]. Unlike Y_2_O_3_, alloys of Ta_2_O_5_ or Nb_2_O_5_ contain lower numbers of cations coordinated to oxygen ions, and therefore increase the phase stability of T-ZrO_2_ [30,32]. Based on these observations, we developed 3Y-TZP co-doped with Nb_2_O_5_ and Ta_2_O_5_, (Y, Nb)-TZP, and (Y, Ta)-TZP. The purpose of the present study was to evaluate the capacity of these LTD-resistant (Y, Nb)-TZP and (Y, Ta)-TZP biomaterials to support osteogenesis, with a view to using them as replacements for current titanium-based dental implant materials.

## 2. Results and Discussion

### 2.1. Results

#### 2.1.1. Surface Analysis of the Titanium and Zirconia Discs

**Figure 1.**
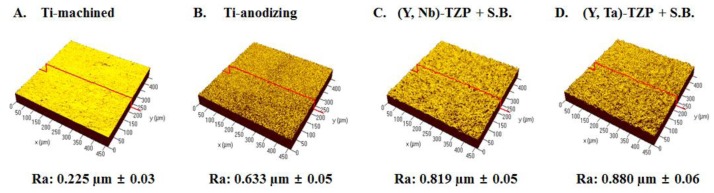
Three-dimensional confocal laser microscopy showing the roughness (*R*_a_) of the examined substrate surfaces. (**A**) Titanium-machined; (**B**) Titanium-anodizing; (**C**) Sandblasted (Y, Nb)-TZP; (**D**) Sandblasted (Y, Ta)-TZP. (S.B.: Sandblasted).

The average roughness values (*R*_a_) of the specimens upon investigation with confocal laser microscopy are shown in Figure 1. The *R*_a_ values of Ti-m and Ti-a were 0.225 μm ± 0.03 (Figure 1A) and 0.633 μm ± 0.05 (Figure 1B), respectively. As previously reported, we increased surface roughness by modifying the surface using anodizing. The average roughness values of (Y, Nb)-TZP and (Y, Ta)-TZP were 0.092 μm ± 0.001 and 0.096 μm ± 0.001 (data not shown). To increase roughness, we sandblasted the zirconia with alumina spraying. Sandblasting with 50-μm alumina (Al_2_O_3_) at 1 bar pressure for 1 min created a rougher surface on the (Y, Ta)-TZP material when compared with (Y, Nb)-TZP (data not shown). To equalize the roughness, (Y, Nb)-TZP was instead subjected to 50 μm alumina (Al_2_O_3_) sandblasting with 2 bar for 1 min. This led to an *R*_a_ of 0.819 μm ± 0.05 for (Y, Nb)-TZP (Figure 1C) and 0.880 μm ± 0.06 for (Y, Ta)-TZP (Figure 1D).

**Figure 2.**
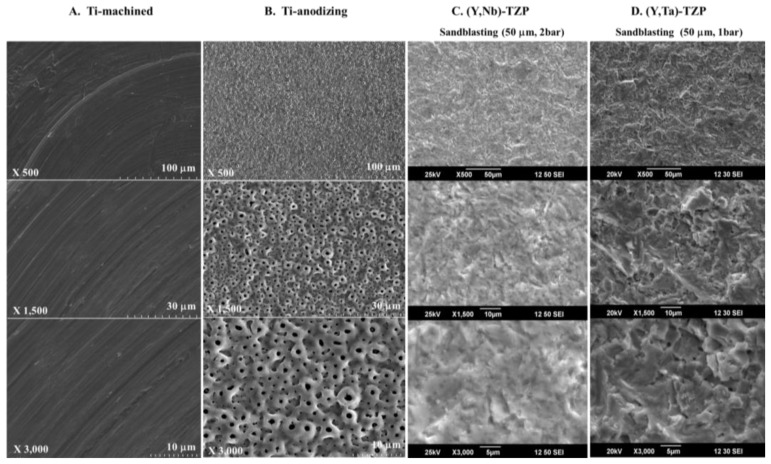
SEM images of Titanium and Zirconia, (**A**) Titanium-machined; (**B**) Titanium-anodizing; (**C**) Sandblasted (Y, Nb)-TZP; (**D**) Sandblasted (Y, Ta)-TZP. Original magnifications are 500, 1500, and 3000×.

The surface morphology of specimens was different. Machined Ti (Ti-m) has grooves because of the grinding operation (Figure 2A). After anodizing, the roughness of Ti significantly increased (Figure 2B). The surface of anodized Ti (Ti-a) was porous with patterned micrographs due to the presence of crystalline structures in the form of rutile and anatase (Figure 2B). The surface morphologies of (Y, Nb)-TZP (Figure 2C) and (Y, Ta)-TZP (Figure 2D) were similar, as each exhibited irregular rough patterns. These results were in good agreement with their average roughness (Figure 1).

#### 2.1.2. Cell Attachment and Morphology

**Figure 3.**
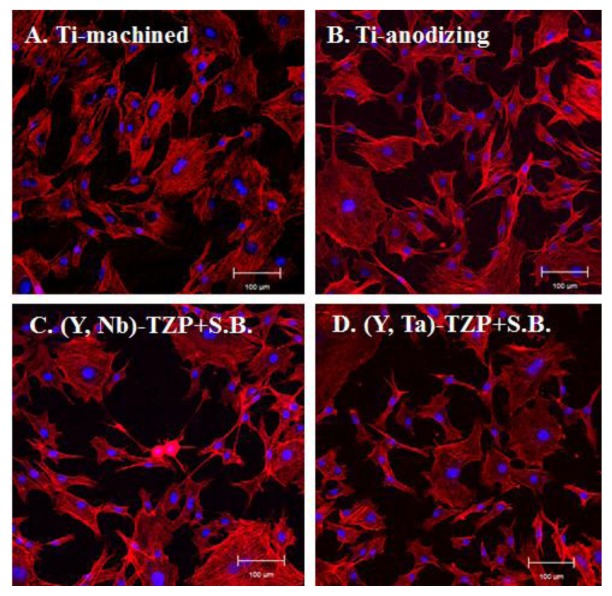
Microscopic observation 24 h after MC3T3-E1 cells were seeded onto the Ti- or Zir-discs. (**A**) Titanium-machined disc; (**B**) Titanium-anodized disc; (**C**) Sandblasted (Y, Nb)-TZP disc; (**D**) Sandblasted (Y, Ta)-TZP disc. Original magnification is 300× and bar = 100 μm.

Twenty-four hours after MC3T3-E1 pre-osteoblast cells were seeded onto the discs, cell attachment and morphology were examined using confocal laser microscopy (Figure 3). Generally, cells that adhered to the polished surface showed a regular, even size morphology (Figure 3A); however, surface roughness produced by anodizing or sandblasting induced slight morphologic irregularities and unequal cell sizes (Figure 3B–D). This appears to be due to the surface roughness caused by uneven grooves. There was little difference in the proportion of cells with flat morphology between samples grown on titanium and those grown on zirconia, regardless of surface roughness.

#### 2.1.3. Cellular Proliferation

**Figure 4.**
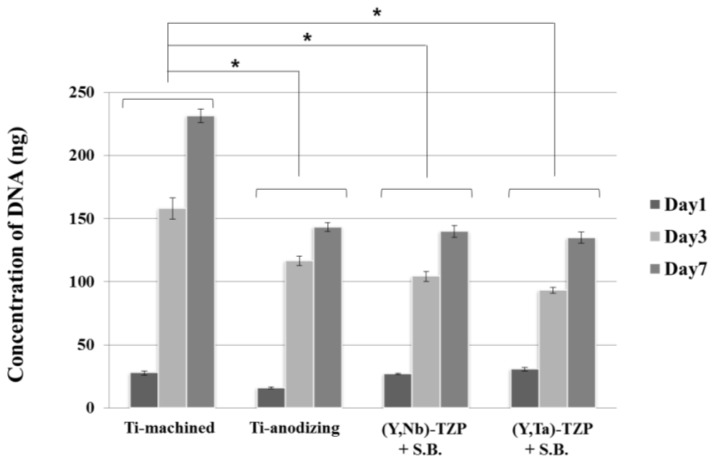
Cell proliferation assay (PicoGreen assay) of MC3T3-E1 cells seeded on the Ti- or Zr-discs at day 1, 3 and 7. Data are expressed as the mean ± SD of three independent experiments. Significance was tested by one-way ANOVA test. * Asterisks indicate *p* < 0.05 against the Ti-machined. (S.B.: Sandblasted).

A PicoGreen assay was performed to examine cellular proliferation. Cells were cultured on the discs and harvested after 1, 3 and 7 day (Figure 4). The proliferation rate increased for the first 3 day, and declined thereafter. Cells on the polished surface (Ti-m) proliferated more rapidly than those on the rough surface discs (Ti-a, (Y, Nb)-TZP and (Y, Ta)-TZP), whereas there was no significant difference between cells grown on Ti-a, (Y, Nb)-TZP and (Y, Ta)-TZP. These results also indicate that the zirconia stabilizers niobium (Nb_2_O_5_) and tantalum (Ta_2_O_5_) are non-toxic to cells and that both (Y, Nb)-TZP and (Y, Ta)-TZP are biocompatible materials.

#### 2.1.4. Osteoblast Differentiation

**Figure 5.**
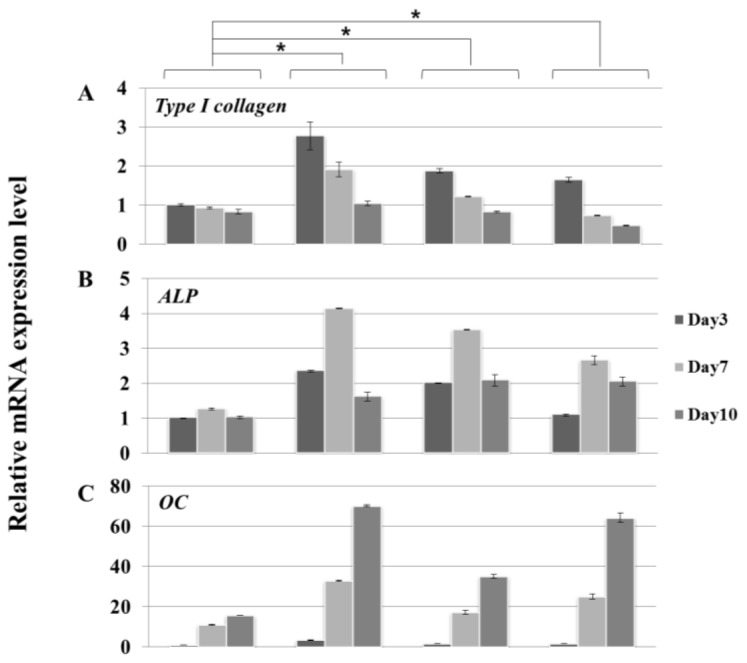
Real-time PCR analysis of MC3T3-E1 cells grown in osteogenic media on Ti- or Zir-discs after 3, 7, and 10 day of culture. (**A**) Type I collagen; (**B**) Alkaline phosphatase (Alp); (**C**) Osteocalcin (Oc). Data are expressed as the mean ± SD of three independent experiments. Significance was tested by one-way ANOVA test. * Asterisks indicate *p* < 0.05 against the Ti-machined.

MC3T3-E1 cells were seeded onto the discs and cultured in osteogenic media. Cells were harvested at 3, 7, and 10 day. We performed molecular profiling of osteoblast differentiation by using real-time PCR (Figure 5). The expression of osteoblast differentiation marker genes, *type I collagen* (Figure 5A), *alkaline phosphatase* (*Alp*) (Figure 5B), and *osteocalcin* (*Oc*) (Figure 5C) was consistent with the differentiation patterns we have previously described [33]. However, there was some variation in the degree of osteoblast differentiation. Cells remained largely undifferentiated on polished surface Ti-m, whereas there was greater differentiation on all the Ti-a, (Y, Nb)-TZP and (Y, Ta)-TZP rough surface discs. The expression profile of differentiation-associated markers was not significantly different between cells grown on the various rough surface discs.

### 2.2. Discussion

Biomaterials for dental implants have to meet the requirement of biocompatibility (e.g., low cellular cytotoxicity, efficient attachment, and support of proliferation and differentiation) [34]. Besides, surface topography, energy and chemical property play an important role in response of cells grown on biomaterials [35,36]. Although many reports have focused on the structural stability and strength of modified zirconia ((Y, Nb)-TZP and (Y, Ta)-TZP) [37,38], few studies have addressed whether the osteogenic response on (Y, Nb)-TZP and (Y, Ta)-TZP is different when compared to traditional titanium implants. In our study, we show that the serious limitation of LTD-dependent destabilization is compensated by addition of either niobium (Nb_2_O_5_) or tantalum (Ta_2_O_5_). As previous studies showed that bone-to-implant surface contact was improved by increasing surface roughness [39], we opted to induce surface roughness by sandblasting with alumina particles (Al_2_O_3_). This process clearly enhanced increased surface roughness, as is also observed following the anodizing procedure. Although this rough surface induced cell morphological irregularities, cell attachment was equivalent between titanium and zirconia, regardless of surface roughness (Figure 3). Orsini and colleagues suggested that morphologic irregularities in sandblasted and acid-etched implants improve initial cell anchorage, thereby providing better osseointegration [40]. Similarly, our data indicated that morphologic irregularities in the rough surfaces (Ti-a, (Y, Nb)-TZP and (Y, Ta)-TZP) (Figure 3) improve the osteogenic response (Figure 5). Cellular proliferation is facilitated by polished surface material (Ti-m) (Figure 4); on the other hand, osteoblast differentiation is predominant in the rough surfaces Ti-a, (Y, Nb)-TZP and (Y, Ta)-TZP), which was confirmed by robust expression of differentiation-associated genes (Figure 5). Osteoblasts are specialized fibroblasts that secrete and mineralize the bone matrix, which contains a high proportion of type I collagen. Osteoblast differentiation proceeds through the three stages of cellular proliferation, matrix maturation, and matrix mineralization. During the initiation stage, genes that encode extracellular matrix proteins (procollagen I and fibronectin) are highly expressed. At the matrix maturation phase (around 7 day culture in the osteogenic media) alkaline phosphatase expression is at its peak, and by the beginning of matrix mineralization, genes encoding osteocalcin, bone sialoprotein, and osteopontin are expressed [33]. Based on the similar osteogenic potential and gene expression profiles we observed between titanium and modified zirconia discs, we are currently exploring strategies to enhance osteogenic potential by using zirconia implants coated with biomolecules such as the pro-osteogenic factors hydroxyapatite or BMP-2 [7,41–45].

## 3. Experimental Section

### 3.1. Specimen Preparation

Pure titanium specimens were prepared in disc shapes (25 mm diameter and 1 mm thickness) through machining (Ti-m, Ti-machined) and treated by anodizing (Ti-a, Ti-anodizing) (OnePlant System, Warrantec Co., Ltd., Seoul, Korea). For the preparation of zirconia specimens, powders of 90.6 mol % ZrO_2_, 5.3 mol % Y_2_O_3_, and 4.1 mol % of Nb_2_O_5_ were mixed for (Y, Nb)-TZP and those of 86.2 mol % ZrO_2_, 7.2 mol % Y_2_O_3_, and 6.4 mol % Ta_2_O_5_ were mixed for (Y, Ta)-YZP. Disc-shaped green compacts (15 mm diameter and 1 mm thickness) were prepared by cold isostatic press of the powder mixtures at 200 MPa and then sintered for 5 h at 1650 °C in air. All zirconia discs were gradually polished and finished with diamond pastes to acquire a mirror-like surface. After polishing, (Y, Ta)-TZP and (Y, Nb)-TZP were sandblasted with 50-μm alumina (Al_2_O_3_) for 1 min with 1 or 2 bar pressure, respectively in order to create a rough surface.

### 3.2. Surface Roughness Assessment

The average surface roughness (*R*_a_) and surface topography were measured using a confocal laser microscope (Carl Zeiss, Oberkochen, Germany). Surface morphology of specimens was observed using a scanning electron microscope (HITACHI S-4700 and JEOL, Tokyo, Japan) after sputter coating with platinum (Pt).

### 3.3. Cell Culture

Mouse pre-osteoblast MC3T3-E1 cells were purchased from ATCC (Manassas, VA, USA) and seeded on the discs and cultured in α-minimal essential medium (α-MEM), which contains 10% fetal bovine serum (FBS) and 1% penicillin/streptomycin. Osteogenic media includes 10 mM β-glycerophosphate and 50 μg/mL ascorbic acid.

### 3.4. Cell Attachment Observation

Confocal microscopy observation was performed. Cells on the discs were fixed in 4% formaldehyde and 4′,6-diamidino-2-phenylindole (DAPI, Invitrogen, Carlsbad, CA, USA) was used for detection of cell nuclei, and Alexa Fluor 568 phalloidin (Invitrogen, Carlsbad, CA, USA) was used for detection of the cytoskeleton. Fluorescence was visualized with a Carl Zeiss LSM700 microscope and analyzed with ZEN2011 software (Carl Zeiss, Oberkochen, Germany).

### 3.5. Cell Proliferation Assay

PicoGreen assay was performed using the Quant-iT PicoGreen assay kit (Invitrogen Ltd., Paisley, UK) at 1, 4, and 7 day after seeding cells on the discs. Cells were washed with PBS and lysed using TE buffer (10 mM Tris-HCl, 1 mM EDTA, pH 7.5). The DNA contents were determined by mixing 100 μL of PicoGreen reagent and 100 μL of DNA sample. Samples were loaded in triplicate and florescence intensity was measured on a GloMax-Multi Detection System machine (Promega, Madison, WI, USA). Florescence intensity was converted into DNA concentration with the DNA standard curve per the manufacturer’s instructions. Values are represented mean ± SD of three independent measurements.

### 3.6. Reverse-Transcription PCR and Quantitative Real-Time PCR

Cells were harvested at 3, 7, and 10 day of osteoblast differentiation and RNA was isolated using QIAzol lysis reagent (QIAGEN, Valencia, CA, USA). The Primescript RT reagent kit for reverse transcription was purchased from TAKARA (Takara Bio, Shiga, Japan). Quantitative real-time PCR was performed with the primer sets for the type I collagen gene, alkaline phosphatase (*Alp*), and osteocalcin (*Oc*) as previously described [33]. Quantitative real-time PCR was performed using Takara SYBR premix Ex Taq (Takara Bio, Shiga, Japan) on Applied Biosystems 7500 Real Time PCR system (Foster City, CA, USA). PCR primers were synthesized by Integrated DNA technology (IDT; Coralville, IA, USA). All samples were run in duplicate, and the relative levels of mRNA were normalized to those of glyceraldehyde-3-phosphate dehydrogenase (*Gapdh*).

### 3.7. Statistical Analysis

All quantitative data are presented as the mean ± SD, each experiment was performed at least three times, and the results from one representative experiment are shown. Significant differences were analyzed using ANOVA-test. A value of *p* < 0.05 was considered statistically significant.

## 4. Conclusions

This *in vitro* study demonstrates that the osteogenic response of cells grown on (Y, Nb)-TZP and (Y, Ta)-TZP substrates is comparable to that observed on titanium, which is widely used in dental implant materials. By compensating the LTD weakness using stabilizers such as niobium oxide (Nb_2_O_5_) or tantalum oxide (Ta_2_O_5_), zirconia is therefore a viable substitute for titanium in terms of both structural stability and biocompatibility. Future studies are now required to determine the *in vivo* efficacy of zirconia implants with respect to osseointegration.
